# Molecular Networking from Volatilome of *Theobroma grandiflorum* (Copoazu) at Different Stages of Maturation Analyzed by HS-SPME-GC-MS

**DOI:** 10.3390/molecules30061209

**Published:** 2025-03-08

**Authors:** Mayrin Valencia, Mónica Pérez-Beltrán, Gerson-Dirceu López, Chiara Carazzone, Paula Galeano Garcia

**Affiliations:** 1 Grupo de Investigación en Productos Naturales Amazónicos (GIPRONAZ), Facultad de Ciencias Básicas, Universidad de la Amazonia, Florencia 180001, Colombia; may.valencia@udla.edu.co; 2 Laboratory of Advanced Analytical Techniques in Natural Products (LATNAP), Chemistry Department, Universidad de los Andes, Bogotá 111711, Colombia; mt.perezb@uniandes.edu.co (M.P.-B.); c.carazzone@uniandes.edu.co (C.C.); 3 Grupo de Investigación en Ciencias y Educación (ICE), Facultad de Ciencias y Humanidades, Universidad de América, Bogotá 111211, Colombia; gerson.dirceu@correounivalle.edu.co; 4 Chemistry Department, Faculty of Natural and Exact Sciences, Universidad del Valle, Cali 760042, Colombia

**Keywords:** *Theobroma grandiflorum*, HS-SPME, GC-MS, volatilomics, molecular networking

## Abstract

*Theobroma grandiflorum* (copoazu) is a plant native to South America, widely cultivated in countries within the Amazon region. Its unique phytochemical composition imparts distinctive organoleptic properties, making it an exotic fruit. In this study, headspace solid-phase microextraction (HS-SPME) combined with gas chromatography–mass spectrometry (GC-MS) was used to identify the volatile organic compounds (VOCs) produced by copoazu. The optimal conditions for sample pretreatment were first determined using a Design of Experiments (DoE) approach. Analysis of the volatile profiles enabled the identification of 96 copoazu VOCs across three ripening stages. Of these, 79 VOCs were classified into chemical compound families using spectral correlation analysis across various libraries and databases, as well as molecular network analysis. Additionally, a volatilomic analysis was conducted to examine the changes in VOCs throughout the ripening process. Molecular network analysis showed that the VOCs emitted by the fruit are linked to the interconversion of compounds, which can be observed through the study of the metabolic pathways. These findings provide a comprehensive analysis of the copoazu volatilome, providing valuable insights into the organoleptic characteristics of this Amazonian fruit. Esters and terpenes such as α-terpineol, *trans*-4-methoxythujane, linalool, 2-methylbutyl butanoate, 3-methylbut-2-enoic acid, 2-methylpentyl ester, and 2-methylpropyl hexanoate were identified as potential biomarkers associated with the copoazu ripening process.

## 1. Introduction

The Amazon region is the world’s largest reservoir of plant genetic resources, home to numerous *Theobroma* species under intensive cultivation [1]. *Theobroma grandiflorum* (copoazu) has attracted significant attention in both domestic and international markets due to its considerable bioeconomic, biotechnological, and horticultural potential [2]. Copoazu cultivation extends across Colombia, Bolivia, Peru, Ecuador, and Brazil [3,4]. The Amazon is the primary production hub, yielding approximately 20,000 tons per year, with pulp production accounting for 3400 tons [4]. In Colombia, copoazu cultivation is concentrated in the Amazonas, Caquetá, Putumayo, and Guaviare regions, where fruit butter is primarily exported to Brazil for use in the cosmetic industry [5].

Copoazu fruit is renowned for its excellent texture, flavor, and aroma, with its economic value derived from the utilization of its pulp, seeds, and peel [6]. The fruit has therapeutic potential attributed to its nutritional and bioactive properties that are beneficial to human health, making it valuable in the food, cosmetic, and pharmaceutical industries. The pulp, mucilaginous in texture and ranging in color from white to creamy yellow, is marketed fresh or processed into products such as jams, ice creams, juices, and yogurt [3]. It is rich in starch, pectin, polysaccharides, and dietary fiber, but primarily insoluble fiber, which enhances the sensory and textural qualities of dairy products [7]. The seeds are a significant source of fats and fatty acids, including oleic, linoleic, and stearic acids, which hold dietary importance [8]. These properties led to the production of a chocolate analog called “cupulate” or “cupulado” [2,9].

Volatile organic compounds (VOCs) are critical quality indicators and commercial attributes that influence the sensory acceptability of fruits and their derived products, particularly in shaping organoleptic characteristics such as flavor and aroma [10]. During ripening, VOCs’ synthesis and regulation are governed by metabolic pathways, making the ripening stage a key determinant of fruit sensory characteristics [11]. A detailed understanding of the volatile profile during ripening enables the identification of compounds with industrial relevance [12], thereby enhancing the value of the fruit and its derivatives as functional food products. Research on copoazu’s VOCs highlights esters as the predominant group, followed by terpenes and alcohols, with notable compounds including ethyl butanoate, ethyl hexanoate, and linalool [13]. Several extraction techniques, such as vacuum distillation, solid-phase extraction, and liquid–liquid extraction, have been employed to analyze such compounds [13,14,15,16,17]. However, these methods often involve multiple steps, the use of contaminating reagents, and lengthy analysis times. In contrast, headspace solid phase microextraction (HS-SPME) offers a solvent-free, cost-effective alternative, with enhanced sensitivity, selectivity, and versatility. These techniques integrate extraction, concentration, desorption, and sampling into single step, streamlining complex sample pretreatment procedures for efficient VOC determination [18,19].

Optimizing HS-SPME factors such as extraction time and temperature, salting-out effects, sample quantity, equilibration time, and temperature typically involves a one-factor-at-a-time (OFAT) approach [20]. However, this method has certain limitations, including the requirement for numerous experiments, the inability to identify factors interactions, and difficulties in achieving a global optimal response. The Design of Experiments (DoE) is a multivariate statistical approach that addresses these limitations by enabling the simultaneous evaluation of individual and interactive variables, reducing time and cost while determining optimal parameter settings [21,22,23]. Response Surface Methodology (RSM), a commonly employed tool in DoE, provides visual representations of optimal values, identifies process inefficiencies, and assesses relationships between factors and responses [24,25].

Gas chromatography–mass spectrometry (GC-MS) is a robust, reproducible, and automated analytical technique widely used to analyze VOCs across various matrices. However, the large volumes of multidimensional data generated by GC-MS analysis can be challenging to process. Therefore, multivariate statistical methods are essential for gaining a deeper understanding of the volatile profiles and condensing these extensive datasets. Volatilomics, an emerging field in omics sciences, focuses on studying plant, fruit, and microbial biochemistry by analyzing VOC composition. This approach provides insights into eco-physiological, environmental, and genetic factors through the study of metabolic networks [26].

This study represents a pioneering effort to comprehensively analyze VOCs in copoazu using HS-SPME-GC-MS, combined with DoE optimization and a volatilomics approach. To the best of our knowledge, this is the first application of a multivariate statistical method to optimize HS-SPME conditions specifically for the analysis of copoazu VOCs, with a particular emphasis on the fruit’s ripening stages. This novel approach enhances VOC extraction and analysis, offering valuable insights into the metabolic changes occurring in fruit ripening. Our findings significantly advance the understanding of copoazu’s volatile profile, enhancing its value across food, cosmetic, and pharmaceutical industries.

## 2. Results and Discussions

### 2.1. Analysis of Volatile Metabolites Using HS-SPME-GC-MS

To optimize the sensitivity and selectivity of HS-SPME for volatile organic compounds (VOCs), various sample pretreatment parameters were evaluated. These included maceration with liquid nitrogen, freezing at −80 °C, lyophilization, salting-out using a 30% NaCl solution (*w*/*v*), and ultrasound treatment. Among these, maceration with liquid nitrogen showed the most significant improvement in chromatographic peak area, with a 37% increase in relative peak area compared to other treatments (Figure S1a). Key VOCs such as linalool (127), ethyl hexanoate (89), 2-methylpentanoic anhydride (68), and 2-methyl-3-buten-2-ol (5) exhibited significant differences (*p* < 0.05) across treatments, with the highest relative area recorded for liquid nitrogen maceration (Figure S1b). This improvement is attributed to the increased matrix surface area and the prevention of VOC loss or enzymatic degradation by freezing [27,28].

Contrary to expectations, lyophilization resulted in decreased sensitivity due to the loss of VOCs during water sublimation. Similarly, the salting-out effect demonstrated inconsistent sensitivity changes, likely influenced by the biological matrix and salt concentration [29]. In this study, the use of salting-out led to reduced chromatographic peak areas. Ultrasound treatment also caused a loss of VOCs, further diminishing the sensitivity of the analysis [30]. As a result, neither lyophilization nor salting-out was included in the sample preparation for VOC analyses. To enhance chromatographic sensitivity, different quantities of copoazu pulp macerated with liquid nitrogen were tested, ranging from 150 to 1200 mg. Figure S2 shows the relative areas of 11 representative chromatographic peaks as a function of sample quantity. Linalool displayed an increase in relative area up to 900 mg but declined at 1200 mg, while other compounds showed no significant variations. Based on these results, 750 mg was chosen as the optimal sample mass, as quantities above 900 mg suppressed VOC volatilization, reducing adsorption onto the SPME fiber—a behavior similarly reported in melon analyses [31]. Pre-equilibrium times of 6, 12, and 18 min were also compared, with no statistically significant differences observed. Consequently, 6 min was selected as the optimal pre-equilibrium time.

### 2.2. DoE

The conditions for extraction time and exposure temperature were optimized using a Design of Experiments (DoE) approach. The relative areas of five key VOCs—2,4,5-trimethyl-1,3-dioxolane, 3-methylbutyl alcohol, hexanal, ethyl 2-methylbutanoate, and 3-methylbutyl ethanoate—across the three ripening stages of copoazu were selected as response variables for the modeling of 3D response surface graphs.

**Figure 1:**
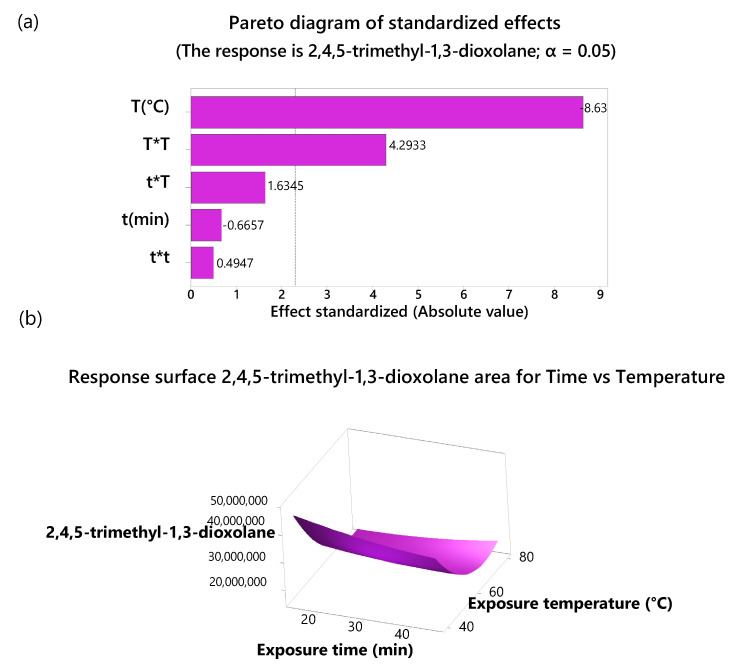
(**a**) Pareto diagram of standardized effects of 2,4,5-trimethyl-1,3-dioxolane. (**b**) Response surface plot of the area of 2,4,5-trimethyl-1,3-dioxolane for the extraction time vs. temperature.

A Pareto chart of standardized effects was generated for each compound (Figure 1a and Figures S3a–S6a). For 2,4,5-trimethyl-1,3-dioxolane (Figure 1a), temperature (T) (40–80 °C) and the quadratic temperature term (T × T) had a statistically significant effect on the response variable (*p* < 0.05). However, the time factor (t) and the interaction between time and temperature (t × T) were not significant. Interestingly, both temperature and time showed negative effects, suggesting that lowering these parameters increased the relative peak area of this compound. Similar trends were observed for 3-methylbutyl alcohol, ethyl 2-methylbutanoate, and 3-methylbutyl ethanoate, where temperature and its quadratic term were the primary significant factors, as shown in the respective Pareto charts (Figures S3a, S5a, and S6a). In contrast, for hexanal (Figure S4a), none of the studied factors had a statistically significant effect on the response variable.

Figure 1b depicts the response surface plot of the area of 2,4,5-trimethyl-1,3-dioxolane as a function of extraction time and temperature. The compound area reached optimal values at 15 min and at 40 °C, with an increasing trend at temperatures below 60 °C. The same behavior was observed in the response surface plot of the areas of 3-methylbutyl alcohol, ethyl 2-methylbutanoate, and 3-methylbutyl ethanoate as a function of the extraction time and temperature (Figures S3b, S5b, and S6b). Hexanal reached the optimal temperature and time values across the entire studied interval (40–80 °C) and (15–45 min), respectively, with a trend of increasing up to 30 min and 60 °C and then decreasing (Figure S4b). The optimal conditions depended on the compound. Therefore, based on the response optimization of the five VOCs, the optimal extraction time and temperature were determined to be 27 min and 40 °C, respectively.

### 2.3. Maturation State Indices

The maturation states of copoazu fruits were determined based on physicochemical analyses. Results indicated a progressive increase in pH and total soluble solids (TSSs) values as maturation advanced (Table S2), consistent with findings in other fruits such as bananas, mangoes, papaya, and blueberries [32,33]. However, the increase in pH was not statistically significant.

#### 2.3.1. Characterization of Copoazu Maturation States by ATR-FTIR

**Figure 2:**
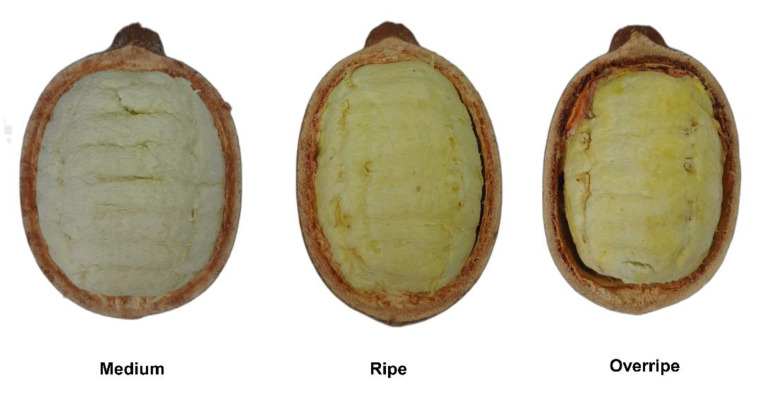
Ripening stages of copoazu. Longitudinal cut of the fruit.

The overlaid FTIR spectra of the three maturation stages (Figure S7) revealed similar overall profiles, with variations in the intensity and presence of certain absorption bands depending on the ripening stage. The absorption band at 1589.34 cm^−1^ increased as the maturation state progressed. This is attributed to carotenoid compounds owing to C-C stretching in highly conjugated double-bond systems [34]. The increased intensity of the band coincided with an increase in carotenoid content, which is responsible for the pale-yellow color of the pulp of the ripe copoazu (Figure 2). Conversely, the bands at 1226.73 and 1712.79 cm^−1^ decrease with copoazu maturation. Bands corresponding to the stretching vibrations of the C-C(O)–C and (C=O⋯H) bonds are related to compounds of the cell wall formed by polysaccharides, such as cutin, phenolic compounds, and pectin [35,36]. During maturation, polysaccharides degrade, intensifying the organoleptic properties of fruits [37].

#### 2.3.2. Carotenoid Analysis by HPLC-DAD

Carotenoids are natural pigments responsible for the yellow, orange, and red coloration in various fruits and microorganisms [38,39]. To investigate the characteristic color of very ripe copoazu pulp, carotenoid profiling was performed using high-performance liquid chromatography (HPLC). Carotenoid identification was carried out by analyzing the absorbances in the UV–visible region, retention times observed on a C30 column, and comparison with spectral data from the literature [40,41]. Table S3 lists the tentative identification of seven copoazu carotenoids, mostly xanthophylls (zeaxanthin, lutein, luteoxanthin, antheraxanthin, neoxanthin, β-carotene, and (all-E)-lutein 3′-O-palmitate), as the major compound with a retention time of 4.48 min [42,43] (Figure S8).

### 2.4. Volatilomics and Molecular Networking Analysis

A total of 96 VOCs were detected and annotated in copoazu across three ripening stages. These compounds were classified as follows: 16 alcohols and polyols, 12 monoterpenoids, three ethers, five 1,3-dioxanes and 1,3-dioxalanes, one dicarboxylic acid derivatives, 29 esters, nine carbonyl compounds (aldehydes, ketones, and acyloins), and 17 unknowns (Table 1). VOCs were identified using the NIST and GNPS libraries, leveraging molecular ions, isotopic distribution, retention time, and fragmentation patterns. GNPS addressed challenges associated with electron ionization (EI) data by employing advanced computational methods such as unsupervised non-negative matrix factorization for effective deconvolution and co-analysis of large datasets [44]. Additionally, terpenoid standards were employed, with confidence levels assigned according to the criteria outlined in Section 2.4. These findings align with previous reports on copoazu VOCs [13,14,15,17]; however, this is the first comparative study on the volatile profiles of copoazu across different ripening stages.

**Figure 3:**
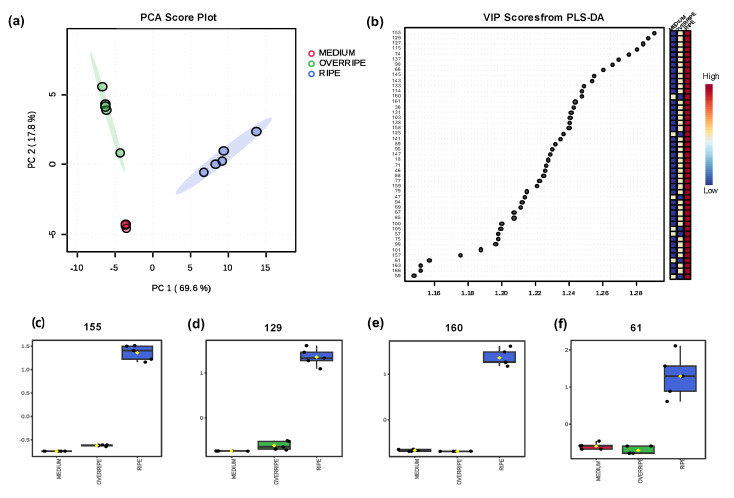
(**a**) PCA score plot for the three maturation stages. (**b**) VIP score plots derived from the PLS-DA analysis, displaying the discriminant features at the three maturation stages, with PLS-DA cross-validation parameters R^2^ = 0.95332 and Q^2^ = 0.90824, indicating good predictive capability. (**c**–**f**) Box plots from the ANOVA of the normalized molecular features α-Terpineol (155), *trans*-4-methoxythujane (129), 1,2-dibutoxyethane (160), and 2-methylbutyl ethanoate (61), respectively. In Figures (**a**–**f**), colors represent ripening stages: *red* for medium, *green* for overripe, and *blue* for ripe. In Figure (**b**), the red-to-blue scale indicates feature intensity across stages.

A volatilomic study explored the variation in VOCs and their correlations across the three ripening stages of copoazu. The study included multivariate statistical analysis and molecular networking to correlate the compounds. Principal component analysis (PCA) was conducted to differentiate the volatile profiles during the maturation stages of copoazu. Figure 3a shows that components (PC1 and PC2) accounted for 87.4% of the variance in the data. Furthermore, analysis revealed three distinct clusters, each corresponding to a specific ripening stage. These clusters demonstrate a significant variation in the volatile profile of Amazonian fruits based on the stage of ripening.

The VIP scores (Figure 3b) displayed 47 significant VOCs that allowed differentiation of the three maturation stages with a VIP > 1. The VOCs were found in high concentrations in the ripe stage, middle concentrations in the overripe stage, and low concentrations in the medium stage. However, the compounds 1,2-dibutoxyethane (160), propyl hexanoate (125), butyl ethanoate (47), β-cis-ocimene (105), 2,2-dimethylpentanoic acid (57), 2-methylbutyl ethanoate (61), and 3-methylbutyl ethanoate (59) were produced in the medium state, reached a maximum concentration in the ripe state, and then decreased in concentration in the overripe state. It is also noticeable that esters and terpenes, such as α-terpineol (155), *trans*-4-methoxythujane (129), and linalool (127), predominated with VIP > 1.28, followed by 2-methylbutyl butanoate (115), 3-methylbut-2-enoic acid, 2-methylpentyl ester (137), unknown (90), vinyl ethanoate (66), and 2-methylpropyl hexanoate (145) with VIP > 1.26. These findings suggest that these VOCs, particularly linalool, α-terpineol, butyl ethanoate, and 3-methylbutyl ethanoate, have potential as biomarkers for copoazu maturation. Although some compounds have been previously identified [13], this study is the first to classify them as maturation stage biomarkers using VIP score analysis.

In the ripe stage, several VOCs exhibited the highest concentrations (relative peak area), distinguishing this stage from the others according to PCA. Key compounds included ethyl butanoate (46), butyl ethanoate (47), 3-methylbut-2-enyl ethanoate (71), unknown (77), butyl butanoate (88), ethyl hexanoate (89), unknown (114), 3-methylbut-2-en-1-yl pivalate (141), propyl hexanoate (125), and 2-ethylphenyl ethanoate (163). Although our study focuses on the analysis of total volatile compounds due to the analysis temperature (60 °C), it is possible to identify semi-volatile compounds that are associated with the fruit aroma. These esters are associated with fruity and sweet flavor profiles [45]. Although terpenoids are present at lower concentrations, they are notable differentiators for this maturation stage. Specifically, β-*trans*-ocimene (105) contributes citrus and floral notes, while *trans*-4-methoxythujane (129) imparts green and woody flavors. The overripe stage is dominated by alcohols, which provide a unique aromatic profile. The odor notes among these compounds—2-methyl-3-buten-2-ol (5), 2-methylpropyl alcohol (7), 1-penten-3-ol (11), 3-pentanol (14), 2-methylbutanol (27), 1-pentanol (31), 2,3-butanediol (44), 3-ethoxy-3-methyl-2-butanone (62), pentan-2-yl propyl carbonate (116), and 2-ethyl-1-hexanol (119)—include oily, herbal, fruity, and sweet profiles [45]. Additionally, the terpenoids 3-thujanone (117), terpinen-4-ol (151), and isothujol (152) share notes of spice, woody, and turpentine [45], further contributing to the distinct aroma profile of the overripe state. In the medium stage, the most concentrated VOCs are 3-methylbutyl alcohol (26), hexanal (45), and 2-methyl-1-butanol (27) with flavor notes of fruity and alcoholic nuances. This study highlights the dynamic influence of ripening on the volatile profile and organoleptic properties of copoazu. The distinct VOC compositions at each stage offer valuable insights into the fruit’s market potential, enabling targeted applications based on flavor and aroma preferences.

**Figure 4:**
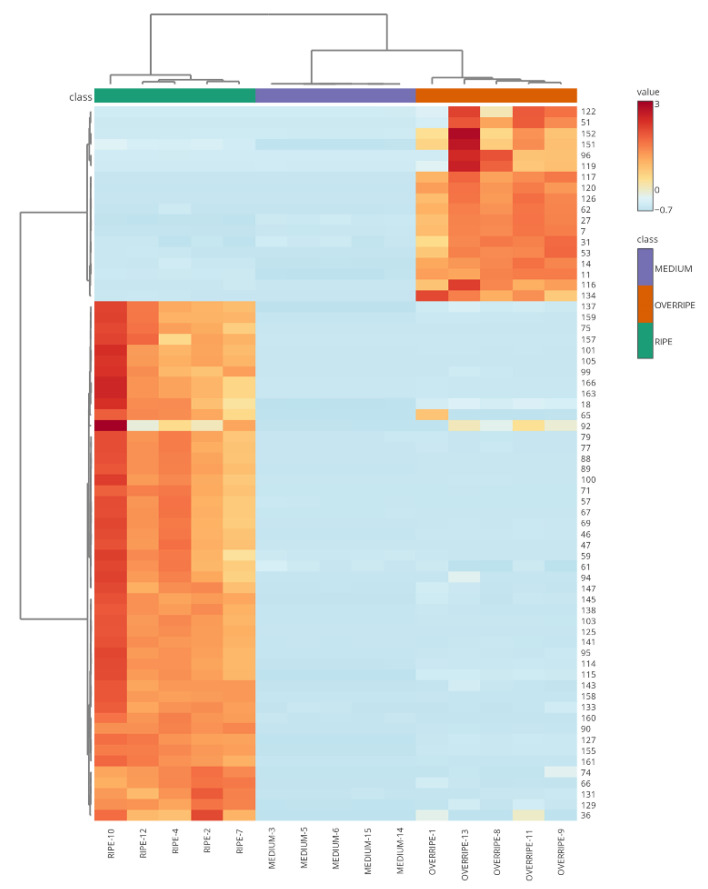
Heatmap analysis of metabolite features associated with VOCs in copoazu. Variation and clustering of the three stages of fruit ripening according to the abundance of detected metabolites. The color spectrum ranging from red to blue indicates the range of high to low signal intensities for each metabolite.

The heatmap in Figure 4 illustrates the clustering patterns of copoazu samples based on the concentrations of 66 significant VOCs (*p*-value > 0.05) at different ripening stages. According to the graph, fruits in the medium and overripe stages of ripening share more similarities, which makes them cluster. On the other hand, ripe fruits had a greater Euclidean distance, indicating a different composition of VOCs compared to the other ripening stages. During medium maturation, late stages of the biosynthetic metabolic pathways of VOCs may occur, that is, volatiles continue to be produced, whose principal function is to protect the growing organ against pathogens and predators [46]. Additionally, compounds present in the medium stage can act as precursors of molecules biosynthesized in the ripe stage, such as 3-methylbutyl alcohol, a precursor in the biosynthesis of esters such as 3-methylbutyl ethanoate [47]. In the overripe stage, alcohols such as 1,3-butanediol (20), 3-methylbutyl alcohol (119), 2-methylbutanol (27), 2-methylpropan-1-ol (7), 1-pentanol (31), 2-ethyl-1-butanol (53), 3-pentanol (14), and 1-penten-3-ol (11) were predominant. This observation is consistent with the report by Klie et al. [48] that ethylene alters fruit metabolism, leading to a climacteric respiratory rise that promotes the biosynthesis of alcoholic substrates that are later used to form esters. In addition, fruits emit alcohol in response to the stress caused by microorganisms or fermentation. For instance, when caimarone grapes metabolize sugars and aliphatic acids, such as tartaric, citric, and malic acids, they generate 2,3-butanediol isomers that have fruity notes or a bread/banana aroma, depending on their levo or meso stereochemistry [49]. In contrast, the ripe stage of copoazu has a higher content of VOCs, particularly esters, which contribute significantly to its characteristic odor. Esterified compounds have been reported to be the most representative of the mature stage in strawberries [50] and purple passion fruit [51]. That is, in the mature stage of the copoazu, VOCs peaked at harvest. Esters can be divided into two groups: carboxylic acid derivatives such as 3-methylbutyl ethanoate (59), 2-methylbutyl ethanoate (61), prenyl ethanoate (71), and ethyl butanoate (46), and fatty acid derivatives such as butyl ethanoate (47), ethyl hexanoate (89), butyl hexanoate (158), 3-methylbutyl hexanoate (166), and butyl butanoate (88), among others. Several of these esters are attributed to fruity, sweet, pineapple, apple, pear, and banana odors [52]. In addition, terpenes such as β-*trans*-ocimene (101), β-*cis*-ocimene (105), and D-Limonene (99) were detected in the ripe stage, whereas 3-thujanone (117) and unknown (151) were found in the overripe stage. According to Nagegowda et al. [53], terpenes undergo different enzymatic modifications, resulting in their vast chemical diversity. Also, Souza et al. [12] suggested that variation in the volatile profile is related to the biosynthesis of terpenoids, which depends on the biosynthesis and cleavage of carotenoids.

**Figure 5:**
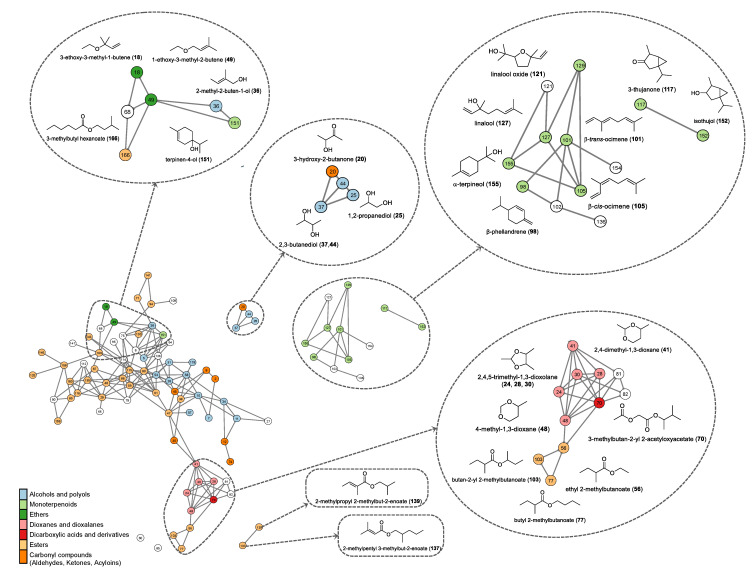
Molecular network from volatilomic profile of the copoazu in three ripening stages. In the molecular network, the biochemical correlation of the compound families is evidenced by the observation that the aldehydes butanal (2) and 3-methylbutanal (8) are precursors of the alcohols butan-1-ol (10) and 3-methylbutanol (26), and the esters resulting from metabolism, such as butyl ethanoate (38). In contrast, in the network dioxane/dioxolane groups are formed by metabolites such as 2,4,5-trimethyl-1,3-dioxolane isomers (24, 28, 30) and 2,4-dimethyl-1,3-dioxane (41). According to Yu et al. [59], this compound is formed by the condensation between acetaldehyde and 2,3-butanediol or ethanol.

Finally, a molecular network of the volatile profile was constructed (Figure 5) to understand the biochemistry of VOCs in copoazu fruit during the three stages of maturation. The network consisted of clusters of compounds that were structurally related to spectral similarity. The network is composed mainly of seven families of compounds: esters (light green), alcohols and polyols (blue), monoterpenoids (green), dioxanes and dioxolanes (pink), carbonyl compounds (aldehydes, ketones, and acyloins) (light orange), and dicarboxylic acids and derivatives (red). These compounds are produced through various metabolic routes; fruits undergo biosynthesis to produce and emit regulated VOCs during development and ripening. For example, aldehydes (2, 8, 45), alcohols (7, 10, 26), and straight-chain esters (89, 125, 158) are derived from fatty acids and amino acids. They have a fresh and fruity aroma and are formed during the ripening period associated with a climacteric increase in respiration [54]. In contrast, branched-chain esters (90, 145) are produced by isoleucine metabolism [55]. C6 and C9 aldehydes are formed via the lipoxygenase-hydroperoxide pathway, in which enzymes oxidize polyunsaturated fatty acids. Hexanal (45) is produced when the substrate is linoleic acid [56].

A study conducted by Liu et al. [55] identified VOCs present in three varieties of apples during fruit development and found that hexanal (45) decreased as the fruits ripened. This decrease is related to ester biosynthesis, because alcohol dehydrogenase reduces aldehydes in the fatty acid and isoleucine degradation pathways to their corresponding alcohols, which are metabolized to esters by the action of alcohol acetyltransferases [57]. The wide variety of esters found in copoazu can be explained by the esterification of linear, branched, or aromatic alcohols with acyl-CoA, which is produced by the oxidative decarboxylation of pyruvate, giving rise to acetyl-CoA. Acyl-CoA produces ethyl esters, whereas acetyl-CoA generates ethanoate esters [58].

Moreover, the molecular network analysis supports the differentiation of VOC profiles across ripening stages (Figure S9). It highlights distinct clustering patterns of VOCs, with some compounds unique to specific stages. For instance, 3-thujanone (117) and isothujol (152) were exclusive to the overripe stage, while 2,2-dimethylpentanoic acid (57) and 2-methylpropyl 2-methylbut-2-enoate (139) were found only in the ripe stage. The medium stage exhibited lower overall VOC concentrations but included compounds such as ethyl butanoate (46) at higher proportions than the other stages.

The correlation between color due to carotenoids and variation in volatile terpenes could be associated with the 2-c-methylerythritol 4-phosphate (MEP) pathway that produces both families of compounds [56]. In this case, the increase in the percentage of terpenes in the ripening stage coincided with the results obtained in the ATR-FTIR spectroscopic analysis, in which spectral bands characteristic of carotenoids were found, in addition to the characterization of these compounds by HPLC. Furthermore, in the molecular network, a well-defined cluster of monoterpenoids (127, 129, 155, 101, etc.) and *trans*-linalool oxide (121) was observed, separated from the main cluster, possibly correlated with the increase in the ripening state of copoazu.

The aromatic composition of fruits also depends on several factors, such as climatology, edaphology, time of harvest, pre- and post-harvest treatments, variety, phenological stage of the fruit [60], storage, fruit condition (whole, sliced, wet, dry), type of analysis (field or laboratory) [19], and VOC extraction technique. Some studies have reported the influence of similar factors on the volatile profiles of sweet pepper [61] and highbush blueberry [33]. Therefore, although most of the VOCs reported in the copoazu were identified; these results could also vary owing to the previously discussed variables. In addition, it was confirmed that the volatile profile of copoazu depended on the maturation stage.

## 3. Materials and Methods

### 3.1. Chemicals and Materials

The HS-SPME-GC-MS analysis utilized an 80 µm × 10 mm Divinylbenzene/Carbon Wide-Range/Polydimethylsiloxane (DVB/CWR/PDMS) fiber (Agilent, Basel, Switzerland) and a manual SPME holder (Supelco, Bellefonte, PA, USA). Liquid nitrogen was sourced from a (Philips System, Son, Netherlands). Sodium chloride was obtained from Sigma-Aldrich, Overijse, Belgium.

Standards for the identification of volatile organic compounds (VOCs) included a terpene mixture (21 components, 2500 µg/mL in hexane) from (DR. EHRENSTORFER, North Charleston, SC, USA) and the Fragrance Allergen Mix A1 from (Supelco, Buchs, Switzerland). HPLC-grade formic acid (≥98%; LiChropur, Supelco, Darmstadt, Germany) and methanol (≥99.9%; Honeywell, Seelze, Germany) were used as the mobile phase and extractant solution for HPLC analysis. Ultrapure water was prepared using a (Heal Force Smart Mini System, Shanghai, China).

### 3.2. Copoazu Collection and Processing

Copoazu fruits were purchased at local market in Paujil, Caquetá, Colombia, (1°37′01.9″ N 75°17′49″ W). Fruits at three maturity stages (medium, ripe, and overripe) were stored at room temperature and transported to the laboratory. Upon arrival, the pulp was separated from the peel and seeds, then frozen at −80 °C. Subsequently, half of the pulp was stored in hermetically sealed bags, while the other half was preserved in borosilicate glass jars for subsequent lyophilization.

### 3.3. VOCs Analysis and Optimization

#### 3.3.1. Analysis of Volatile Metabolites Using HS-SPME-GC-MS

For the analysis, 750 mg of copoazu pulp, previously grounded with liquid nitrogen, was weighed and transferred to a 20 mL headspace vial. The vial was tightly sealed with a silicon septum and pre-equilibrated in an oil bath at 60 °C for 6 min. An 80 µm × 10 mm DVB/CWR/PDMS fiber was then inserted into the vial’s headspace to extract the volatile metabolites for 30 min at 60 °C. Subsequently, the fiber was desorbed at 250 °C for 5 min in the injection port of the GC-MS in splitless mode, following previously reported conditions [62].

VOCs were separated using a capillary GC column composed of 5% Phenyl/95% Dimethyl Polysiloxane (30 m × 0.25 mm × 0.25 μm; SGE, Austin, TX, USA) and analyzed with a Gas Chromatograph HP 6890 Series equipped with an Agilent Mass Selective Detector 5973 (Agilent Technologies, Palo Alto, CA, USA). The optimized heating ramp began at 35 °C for 0.5 min, then increased at 1 °C/min to 40 °C, raised to 60 °C at 1.5 °C/min, finally ramped at 5 °C/min to 120 °C, and held for 1 min. High-purity helium (99.999%) was used as carrier gas at a flow rate of 1.3 mL/min. The mass spectrometer was operated under the following conditions: electronic ionization mode; ion source temperature, 230 °C; electron energy, 70 eV; quadrupole mass range, 30–300 amu; transmission line temperature, 230 °C; detector voltage, 1.4 kV; and quadrupole temperature, 150 °C. VOCs were tentatively identified by comparing the mass spectra of detected metabolites with those in the NIST 17.0 and GNPS libraries. Standards, including a terpene mixture (Terpene mixture1, 21 components, 2500 µg/mL in hexane) and an allergen mix (Fragrance Allergen Mix A1), were used at a concentration of 600 ppm for reference. The relative percentage content of each compound in copoazu samples at three ripening stages was calculated using the peak relative area normalization method.

#### 3.3.2. Optimization of Analysis Conditions Using Design of Experiments

To optimize the extraction of VOCs from copoazu, a 2 × 3 factorial design was applied, with the exposure time of SPME fiber (15, 30, and 45 min) and exposure temperature of SPME fiber (40, 60, and 80 °C) as the independent variables. The response variable was the relative area of peaks observed in the three maturity states of the copoazu pulp. The experimental design was conducted using the statistical software Minitab (version 19.1) [23]. Table S1 presents the randomized conditions generated by the software, along with the corresponding number of experiments performed.

#### 3.3.3. Quality Control

To guarantee the accuracy of the analysis and reliability of the results, rigorous quality control (QC) measures were implemented. During the sample preparation, QC was performed as follows: blank column analysis for monitoring carry-over and blank sample analysis for monitoring the memory effect on SPME fibers. Column and fiber blanks were performed before initiating metabolite analysis. Additionally, intermediate fiber blanks were conducted during and after the analysis sequence to ensure consistent and accurate results.

### 3.4. Data Treatment, Metabolite Annotation, and Molecular Networking

GC-MS data were initially collected using the Agilent MSD ChemStation D.02.00.275 software (.D format) and converted via the Agilent GC MSD translator. Data were then transformed into mzML format using ProteoWizard’s msConvert software (version 3.0) [63] and uploaded to MassIVE https://massive.ucsd.edu (accessed on 26 February 2024). Subsequently, the MSHub deconvolution workflow [44] was applied to generate a spectrum file (.mgf) and a quantification table (.csv), which were directly used as input for GNPS’s molecular-library search-GC workflow.

Processed data were exported to the Agilent MassHunter Quantitative B.04.00 software for integration. The data were filtered for reproducibility by calculating the coefficient of variation (CV) of the signal intensities across samples. Molecular features with a CV > 30% were excluded. Data exhibiting consistent absence or presence across groups were retained. Autoscaling normalization and weight-based correction factors were applied for statistical analysis [64].

An unsupervised principal component analysis (PCA) was conducted to verify analytical reproducibility and sample distribution. To identify statistically significant molecular features across ripening stages, both univariate (UVA) and multivariate (MVA) statistical analyses were performed using the MetaboAnalyst 6.0 server [65].

Metabolites were annotated based on a range of criteria, including database searches, molecular ion analysis, isotopic distribution, retention time (RT), MS spectra, fragmentation pathways, and comparison with reference standards. The annotation confidence levels were reported from 0 to 4 [66]. For the ID levels, 0 corresponds to an unambiguous 3D structure; 1 to a confident 2D structure or reference standard match; 2 to a likely structure or coincidence with the literature spectra; 3 to a possible structure, verified with molecular formula; and 4 to an unknown feature or a basic match with the libraries.

The databases used included GNPS (Global Natural Products Social Molecular Networking, version 30; https://gnps.ucsd.edu (accessed on 24 December 2024) [29], NIST (National Institute of Standards and Technology, library 2.2 version 2014), and FlavorDB https://cosylab.iiitd.edu.in/flavordb/ (accessed on 27 July 2024) [45] to identify flavor-related molecules.

A molecular network was constructed using GNPS’s with the Library Search/Molecular Networking GC workflow. Nodes were linked if their spectrum similarity cosine score was <0.70 with at least six matched peaks. Each node’s connections were limited to its top 10 most similar nodes. Families were restricted to a maximum of 100 nodes by removing the lowest-scoring edges. VOCs were annotated based on GNPS library matches with a balance score threshold of ≥65% to ensure high-confidence identifications [67].

The resulting network file (.graphml) was visualized in Cytoscape (version 3.10.1) [68]. Low-confidence matches were manually removed from the network. Annotated compounds were classified using ClassyFire [69].

### 3.5. Characterization of Copoazu Maturation Stages

#### 3.5.1. pH and Total Soluble Solids (TSSs)

The pH of the fruit was measured by dissolving 1 g of pulp in 5 mL of distilled water using a potentiometer (PHS-3BW Microprocessor/mV/Temperature Meter, Shanghai, China). Total soluble solids (TSS) in the pulp juice were evaluated with a refractometer (Eloptron, Schmidt + Haensch GmbH & Co., Berlin, Germany). All measurements were performed in triplicate for each ripening stage.

#### 3.5.2. ATR-FTIR Analysis

Lyophilized and finely powdered pulp samples were analyzed directly on the attenuated total reflectance (ATR) crystal of a Fourier Transform Infrared Spectroscopy (FTIR) instrument (IR Tracer-100, SHIMADZU, Madison, WI, USA) equipped with LabSolutions Ver.2.33 software. Spectra were collected within the range of 400–4000 cm⁻^1^. Both the background and the sample were scanned 32 times at a resolution of 8 cm⁻^1^. Air was used as the background prior to each sample analysis [70].

#### 3.5.3. Carotenoids Analysis by HPLC-DAD 

Carotenoids were extracted from 250 mg of lyophilized copoazu pulp using 4 mL of a methanol:formic acid solution (99:1; *v*/*v*). The mixture was ultrasonicated (Bransonic 1510R-MT, Danbury, CT, USA) for 20 min, and subsequently centrifuged (Thermo Electron, IEC Centra CL3, Needham, MA, USA) at 3500 rpm for 10 min, according to a previous report by López et al. [71]. The resulting supernatant was evaporated to dryness. A stock solution at 10.000 ppm of the dried extract was prepared in methanol, filtered through a 0.45 µm PTFE membrane, and subjected to HPLC analysis. HPLC analysis was performed using an (ultrahigh-performance liquid chromatographer Dionex UltiMate 3000 equipped with a diode array detector (DAD). The raw data were acquired and processed using Xcalibur 4.3 software (Thermo Scientific, San Jose, CA, USA). Chromatographic separation was carried out under previously published conditions [72] at 30 °C. A 10 µL sample was injected into a YMC-C30 column (150 × 4.6 mm i.d., 3 µm particle size; YMC America, Inc., Devens, MA, USA) protected with a SecurityGuard Cartridge Phenomenex C18 (4 × 2 mm, 3 µm particle size) pre-column. The mobile phase consisted of 400 mg/L of ammonium acetate dissolved in a methanol:methyl tert-butyl ether:water mixture (80:18:2 *v*/*v*/*v*, for solution A and 8:89:3 *v*/*v*/*v*, for solution B). The total run time was 40 min. The DAD recorded absorbances across the entire UV–vis spectrum (240–600 nm), with carotenoids’ characteristic absorbances specifically extracted at 450 nm. Experiments were realized with three biological replicates.

#### 3.5.4. Statistical Analysis for the Characterization of Maturation States of Copoazu

Univariate analyses were conducted in duplicate, and the results are presented as mean ± standard deviation (SD). Data were analyzed using an analysis of variance (ANOVA) performed with InfoStat software (version 2020). For multiple comparisons of means, a generalized linear mixed model (GLMM) was employed, with a *p*-value threshold of ≤0.05 for statistical significance. The LSD–Fisher test was applied as a post hoc method for pairwise comparisons [73].

## 4. Conclusions

This study presents a comprehensive analysis of the volatilome of *Theobroma grandiflorum* at three different maturation stages (medium, ripe, and overripe) using HS-SPME-GC-MS analysis and molecular networks. Additionally, the use of DoE for optimizing sample preparation and SPME conditions helped develop a simple extraction process, improving the sensitivity of VOC analysis. The conditions for the extraction of VOCs were achieved using 750 mg of the sample grounded with liquid nitrogen and an SPME fiber pre-equilibration time of 6 min. In addition, by DoE, the optimal exposition time and temperature were determined to be 27 min and 40 °C, respectively.

The 96 VOCs exhibited variable abundances throughout the different maturation stages. The volatile profiles were analyzed using multivariate analyses, such as PCA and PLS-DA, which enabled the identification of specific VOCs that could serve as potential biomarkers for monitoring the ripening process of copoazu. Notable VOCs include α-terpineol, *trans*-4-methoxythujane, linalool, 2-methylbutyl butanoate, 3-methylbut-2-enoic acid, 2-methylpentyl ester, unknown, vinyl ethanoate, and 2-methylpropyl hexanoate. Additionally, the ripe stage has emerged as a transitional stage in VOC production, with a potential biomarker showing a decrease in abundance in the overripe stage and absence in the medium stage due to the presence of precursors. Compounds that differentiated the odor of copoazu at different stages of maturation were identified. In the mature stage, the fruity, sweet, citric, and floral odors are attributed to esters and terpenes, whereas in the overripe stage, the alcohols are related to the herbal, woody, and turpentine odors.

On the other hand, the molecular network analysis enabled the visualization of structural relationships between various groups of volatile compounds, including esters, alcohols, monoterpenoids, and carbonyl compounds. The molecular network of VOCs evidenced the chemical similarities observed in the different families of compounds that correlated due to their biosynthetic pathways. The findings of this study provide a solid foundation for future studies. Further exploration of the biosynthetic mechanisms underlying VOCs production and the application of biomarker monitoring to optimize copoazu harvesting and processing are possible prospective applications in the food industry.

## Figures and Tables

**Table 1:** VOCs identified in the copoazu in three ripening stages.

GNPS Scan Number	t_R_	Metabolite	Formula	Confidence Level *	Identification	ClassyFire (SubClass)
5	2.30	2-methylbut-3-en-2-ol	C_5_H_10_O	3	NIST	Alcohols and polyols
7	2.47	2-methylpropan-1-ol	C_4_H_10_O	2	GNPS, NIST	Alcohols and polyols
10	2.93	butan-1-ol	C_4_H_10_O	2	GNPS, NIST	Alcohols and polyols
11	3.22	pent-1-en-3-ol	C_5_H_10_O	2	GNPS, NIST	Alcohols and polyols
14	3.53	pentan-3-ol	C_5_H_12_O	2	GNPS, NIST	Alcohols and polyols
25	4.26	propane-1,2-diol	C_3_H_8_O_2_	3	GNPS	Alcohols and polyols
26	4.35	3-methylbutan-1-ol	C_5_H_12_O	2	GNPS, NIST	Alcohols and polyols
27	4.44	2-methylbutan-1-ol	C_5_H_12_O	2	GNPS, NIST	Alcohols and polyols
31	5.41	pentan-1-ol	C_5_H_12_O	3	NIST	Alcohols and polyols
34	5.53	pent-2-en-1-ol	C_5_H_10_O	2	GNPS, NIST	Alcohols and polyols
36	5.68	2-methylbut-2-en-1-ol	C_5_H_10_O	2	GNPS, NIST	Alcohols and polyols
37	5.79	2,3-butanediol (Isomer I)	C_4_H_10_O_2_	2	GNPS, NIST	Alcohols and polyols
44	6.27	2,3-butanediol (Isomer II)	C_4_H_10_O_2_	3	GNPS	Alcohols and polyols
53	8.58	2-ethylbutan-1-ol	C_6_H_14_O	2	GNPS, NIST	Alcohols and polyols
58	10.25	hexan-1-ol	C_6_H_14_O	2	GNPS, NIST	Fatty alcohols
119	24.12	2-ethyl-1-hexanol	C_8_H_18_O	4	GNPS	Alcohols and polyols
2	2.15	butanal	C_4_H_8_O	3	NIST	Carbonyl compounds
8	2.78	3-methylbutanal	C_5_H_10_O	2	GNPS, NIST	Carbonyl compounds
12	3.46	pentan-3-one	C_5_H_10_O	3	NIST	Carbonyl compounds
20	3.74	3-hydroxy-2-butanone	C_4_H_8_O_2_	3	NIST	Carbonyl compounds
35	5.60	3-methylpentan-2-one	C_6_H_12_O	2	GNPS, NIST	Carbonyl compounds
45	6.50	hexanal	C_6_H_12_O	3	GNPS	Carbonyl compounds
74	15.11	5-hydroxy-2,7-dimethyl-4-octanone	C_10_H_20_O_2_	3	GNPS	Carbonyl compounds
84	18.77	6-methyl-5-hepten-2-one	C_8_H_14_O	2	GNPS, NIST	Carbonyl compounds
131	25.49	nonanal	C_9_H_18_O	3	NIST	Carbonyl compounds
18	3.65	3-ethoxy-3-methylbut-1-ene	C_7_H_14_O	3	NIST	Ethers
49	7.75	1-ethoxy-3-methylbut-2-ene	C_7_H_14_O	2	GNPS, NIST	Ethers
64	11.30	3-butoxy-2-methylbut-1-ene	C_9_H_18_O	4	NIST	Ethers
87	19.18	β-myrcene	C_10_H_16_	1	NIST, STD	Monoterpenoids
98	21.45	β-phellandrene	C_10_H_16_	3	GNPS	Monoterpenoids
99	21.45	limonene	C_10_H_16_	1	GNPS, NIST, STD	Monoterpenoids
101	22.27	β-*trans*-ocimene	C_10_H_16_	1	GNPS, NIST, STD	Monoterpenoids
105	22.82	β-*cis*-ocimene	C_10_H_16_	1	GNPS, NIST, STD	Monoterpenoids
117	23.98	3-thujanone	C_10_H_16_O	3	GNPS	Monoterpenoids
121	24.68	*cis*-linalool oxide	C_10_H_18_O_2_	3	GNPS, NIST	Tetrahydrofurans
120	24.68	*trans*-linalool oxide	C_10_H_18_O_2_	2	NIST	Tetrahydrofurans
127	25.35	linalool	C_10_H_18_O	1	GNPS, NIST, STD	Monoterpenoids
129	25.44	*trans*-4-methoxythujane	C_11_H_20_O	3	GNPS	Monoterpenoids
152	28.30	isothujol	C_10_H_18_O	3	GNPS	Monoterpenoids
155	28.75	α-terpineol	C_10_H_18_O	1	GNPS, NIST, STD	Monoterpenoids
24	4.15	2,4,5-trimethyl-1,3-dioxolane (Isomer I)	C_6_H_12_O_2_	2	GNPS, NIST	1,3-dioxolanes
28	4.88	2,4,5-trimethyl-1,3-dioxolane (Isomer II)	C_6_H_12_O_2_	2	GNPS, NIST	1,3-dioxolanes
30	5.33	2,4,5-trimethyl-1,3-dioxolane (Isomer III)	C_6_H_12_O_2_	2	GNPS, NIST	1,3-dioxolanes
41	6.12	2,4-dimethyl-1,3-dioxane	C_6_H_12_O_2_	2	GNPS, NIST	1,3-dioxanes
48	7.57	4-methyl-1,3-dioxane	C_5_H_10_O_2_	2	GNPS, NIST	1,3-dioxanes
54	8.96	2,4,6-trimethyl-1,3-dioxane	C_7_H_14_O_2_	3	NIST	1,3-dioxanes
70	13.38	3-methylbutan-2-yl 2-acetyloxyethanoate	C_9_H_16_O_4_	3	GNPS	Dicarboxylic acids and derivatives
29	5.10	ethyl 2-methylpropanoate	C_6_H_12_O_2_	2	GNPS, NIST	Carboxylic acid derivatives
46	6.64	ethyl butanoate	C_6_H_12_O_2_	2	GNPS, NIST	Carboxylic acid derivatives
47	7.25	butyl ethanoate	C_6_H_12_O_2_	2	GNPS, NIST	Fatty acid esters
56	9.07	ethyl 2-methylbutanoate	C_7_H_14_O_2_	2	GNPS, NIST	Fatty acid esters
59	10.67	3-methylbutyl ethanoate	C_7_H_14_O_2_	2	GNPS, NIST	Carboxylic acid derivatives
61	10.83	2-methylbutyl ethanoate	C_7_H_14_O_2_	2	GNPS, NIST	Carboxylic acid derivatives
69	12.26	ethyl pentanoate	C_7_H_14_O_2_	3	GNPS	Fatty acid esters
71	13.87	3-methylbut-2-enyl ethanoate	C_7_H_12_O_2_	2	GNPS, NIST	Carboxylic acid derivatives
86	18.92	ethyl 5-hexenoate	C_8_H_14_O_2_	2	GNPS, NIST	Fatty acid esters
88	19.62	butyl butanoate	C_8_H_16_O_2_	2	GNPS, NIST	Fatty acid esters
89	19.95	ethyl hexanoate	C_8_H_16_O_2_	2	GNPS, NIST	Fatty acid esters
94	20.67	ethyl-4-hexenoate	C_8_H_14_O_2_	2	GNPS, NIST	Fatty acid esters
100	22.10	pent-4-en-1-yl butanoate	C_9_H_16_O_2_	3	GNPS	Fatty acid esters
103	22.53	butan-2-yl 2-methylbutanoate	C_9_H_18_O_2_	3	GNPS, NIST	Fatty acid esters
115	23.43	2-methylbutyl butanoate	C_9_H_18_O_2_	2	GNPS, NIST	Fatty acid esters
116	23.56	pentan-2-yl propyl carbonate	C_9_H_18_O_3_	3	GNPS	Carbonic acid diesters
125	25.20	propyl hexanoate	C_9_H_18_O_2_	2	GNPS, NIST	Fatty acid esters
137	26.26	2-methylpentyl 3-methylbut-2-enoate	C_11_H_20_O_2_	3	GNPS	Fatty acid esters
139	26.77	2-methylpropyl 2-methylbut-2-enoate	C_9_H_16_O_2_	3	GNPS	Fatty acid esters
141	27.05	3-methylbut-2-en-1-yl pivalate	C_10_H_18_O_2_	2	GNPS, NIST	Carboxylic acid derivatives
143	27.38	4-methylpentyl butanoate	C_10_H_20_O_2_	3	GNPS	Fatty acid esters
145	27.44	2-methylpropyl hexanoate	C_10_H_20_O_2_	2	GNPS, NIST	Fatty acid esters
157	28.81	ethyl oct-4-enoate	C_10_H_18_O_2_	3	NIST	Fatty acid esters
158	28.88	butyl hexanoate	C_10_H_20_O_2_	2	GNPS, NIST	Fatty acid esters
159	29.11	ethyl octanoate	C_10_H_20_O_2_	2	GNPS, NIST	Fatty acid esters
160	30.24	1,2-dibutoxyethane	C_10_H_18_O_4_	2	GNPS, NIST	Fatty acid esters
161	30.56	ethyl 2-phenylethanoate	C_10_H_12_O_2_	2	GNPS, NIST	Benzene and substituted derivatives
163	30.60	2-ethylphenyl ethanoate	C_10_H_12_O_2_	2	GNPS, NIST	Phenol esters
166	30.81	3-methylbutyl hexanoate	C_11_H_22_O_2_	2	GNPS, NIST	Fatty acid esters
57	9.27	2,2-dimethylpentanoic acid	C_7_H_14_O_2_	3	GNPS	Fatty acids and conjugates
63	11.20	ethenylbenzene	C_8_H_8_	3	GNPS	Styrenes
109	23.00	2,7-dimethyl-2,6-octadiene	C_10_H_18_	2	GNPS, NIST	Branched unsaturated hydrocarbons
33	5.41	unknown	-	4	-	unknown
43	6.27	unknown	-	4	-	unknown
51	7.80	unknown	-	4	-	unknown
65	11.30	unknown	-	4	-	unknown
75	15.34	unknown	-	4	-	unknown
77	15.34	unknown	-	4	-	unknown
79	16.16	unknown	-	4	-	unknown
80	16.33	unknown	-	4	-	unknown
90	20.07	unknown	-	4	-	unknown
92	20.38	unknown	-	4	-	unknown
95	20.80	unknown	-	4	-	unknown
114	23.30	unknown	-	4	-	unknown
122	25.16	unknown	-	4	-	unknown
126	25.30	unknown	-	4	-	unknown
133	25.53	unknown	-	4	-	unknown
147	27.76	unknown	-	4	-	unknown
151	28.25	unknown	-	4	-	unknown

* Confidence levels, 1 to a confident 2D structure or reference standard match; 2 to a likely structure or coincidence with the literature spectra; 3 to a possible structure, confirmation with molecular formula; and 4 to an unknown feature or a basic match with libraries. The different compound families were color-coded to facilitate correlation in Molecular network from volatilomic profile of the copoazu in three ripening stages. Alcohols and polyols are represented in blue, monoterpenoids in green, esters in light green, dioxanes and dioxolanes in pink, and carbonyl compounds—such as aldehydes, ketones, and acyloins—in light orange. Dicarboxylic acids and their derivatives are shown in red, other compounds in gray, and unknowns remain colorless.

## Data Availability

All data from this research are available in this paper and Supplementary Materials including the web links for the MSHub/GNPS jobs generated, which can be found on https://gnps.ucsd.edu/ProteoSAFe/status.jsp?task=c047070e264a497984029f6890f194c3 accessed on 7 November 2024, https://gnps.ucsd.edu/ProteoSAFe/status.jsp?task=e865a49261d948cdae02d41081b6f2e8 accessed on 20 November 2023.

## Supplementary Materials

The following supporting information can be downloaded at: https://www.mdpi.com/article/10.3390/molecules30061209/s1. Figure S1: Influence of sample treatment on the total area of 6 majority VOCs. (a) Total area and (b) area of each compound. The results are expressed as the mean ± SEM of duplicate analyses. Different letters in each composite indicate a significant difference at *p* ≤ 0.05, as determined by the LSD-Fisher test. Figure S2: Optimization of the sample amount. Results were expressed as the mean ± SEM of duplicate analysis. Different letters in each compound indicate a significant difference at *p* ≤ 0.05, as determined by the LSD-Fisher test. Figure S3: (a) Pareto plot of standardized effects for 3-methylbutyl alcohol and (b) area response surface of isoamyl alcohol over time vs. extraction temperature. Figure S4: (a) Pareto plot of standardized effects for hexanal and (b) hexanal area response surface for time vs. extraction temperature. Figure S5: (a) Pareto plot of standardized effects for ethyl 2-methylbutanoate and (b) area response surface of ethyl 2-methylbutanoate over time vs. extraction temperature. Figure S6: (a) Pareto plot of standardized effects for 3-methylbutyl ethanoate and (b) area response surface of isoamyl acetate for time versus extraction temperature. Figure S7: FTIR spectra of ripening stages of copoazu. Medium-ripening stage (black), ripe (red), and very ripe (green). Figure S8: HPLC-DAD chromatogram of carotenoids in copoazu. Figure S9: Molecular network from volatilomic profile of the copoazu in three ripening stages. In the molecular network shown the representation of the absolute intensity of the mass spectra at each stage of maturation according to compounds. Table S1: 2 × 3 factorial design. Table S2: Physicochemical parameters of copoazu fruit at different stages of ripening. Table S3: Tentative identification of seven copoazu carotenoids.
