# Supplementary material for: Molecular Networking from Volatilome of Theobroma grandiflorum (Copoazu) at Different Stages of Maturation Analyzed by HS-SPME-GC-MS
Source: Molecules. 2025 Mar 8;30(6):1209. doi: 10.3390/molecules30061209 (PMC11944471; doi:10.3390/molecules30061209)
Supplement: Supplementary file 1 [file molecules-30-01209-s001.zip › molecules-3443558-supplementary.pdf]

MATERIAL SUPPLEMENTARY

Molecular networking from volatilome of *Theobroma grandiflorum* (Copoazu) at different stages of maturation analyzed by HS-SPME-GC-MS

Mayrin Valencia[1,2,3], Mónica Pérez-Beltrán[2], Gerson-Dirceu López[3,4], Chiara Carazzone[2], Paula Galeano,[1,2*]

[1] Grupo de Investigación en Productos Naturales Amazónicos -GIPRONAZ- Facultad de Ciencias Básicas, Universidad de la Amazonia, Florencia, 180001, Colombia.

[2] Laboratory of Advanced Analytical Techniques in Natural Products (LATNAP), Chemistry Department, Universidad de los Andes, Bogotá 111711, Colombia.

[3] Grupo de Investigación en Ciencias y Educación (ICE), Facultad de Ciencias y Humanidades, Universidad de América, Bogotá 111211, Colombia.

[4] Chemistry Department, Faculty of Natural and Exact Sciences, Universidad del Valle, Cali 760042, Colombia.

*Correspondence: p.galeano@udla.edu.co

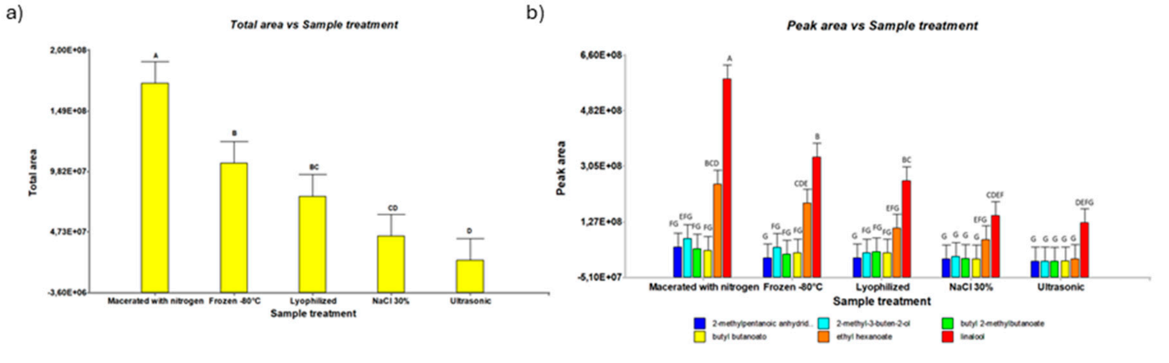

Figure S1. Influence of sample treatment on the total area of 6 majority VOCs. a) Total area and b) area of each compound. The results are expressed as the mean ± SEM of duplicate analyses. Different letters in each composite indicate a significant difference at $p \leq 0.05$, as determined by the LSD-Fisher test.

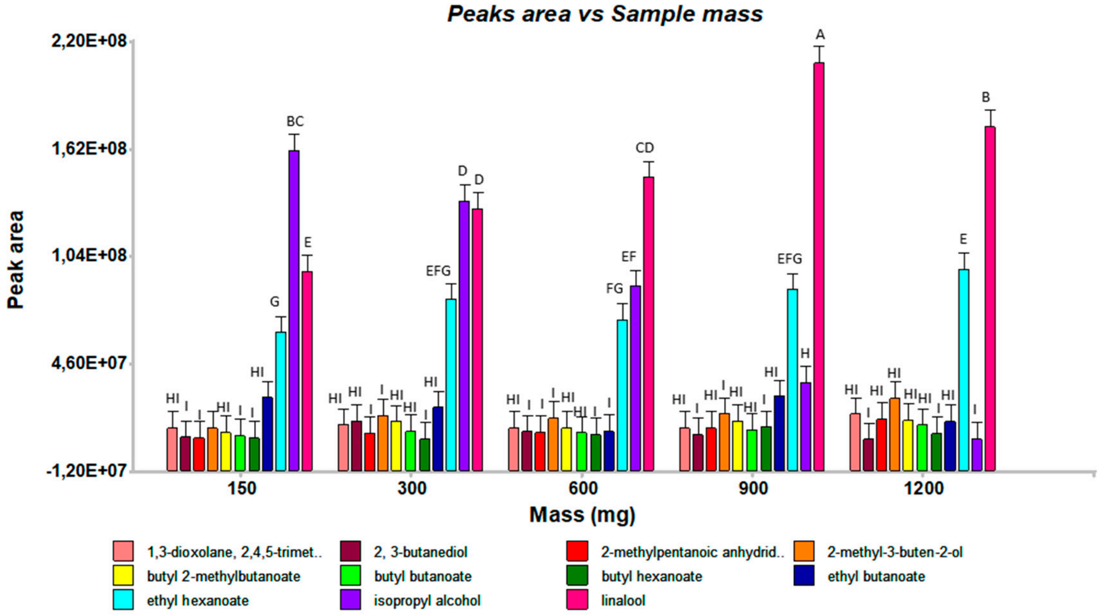

Figure S2. Optimization of the sample amount. Results were expressed as the mean ± SEM of duplicate analysis. Different letters in each compound indicate a significant difference at p ≤ 0.05, as determined by the LSD-Fisher test.

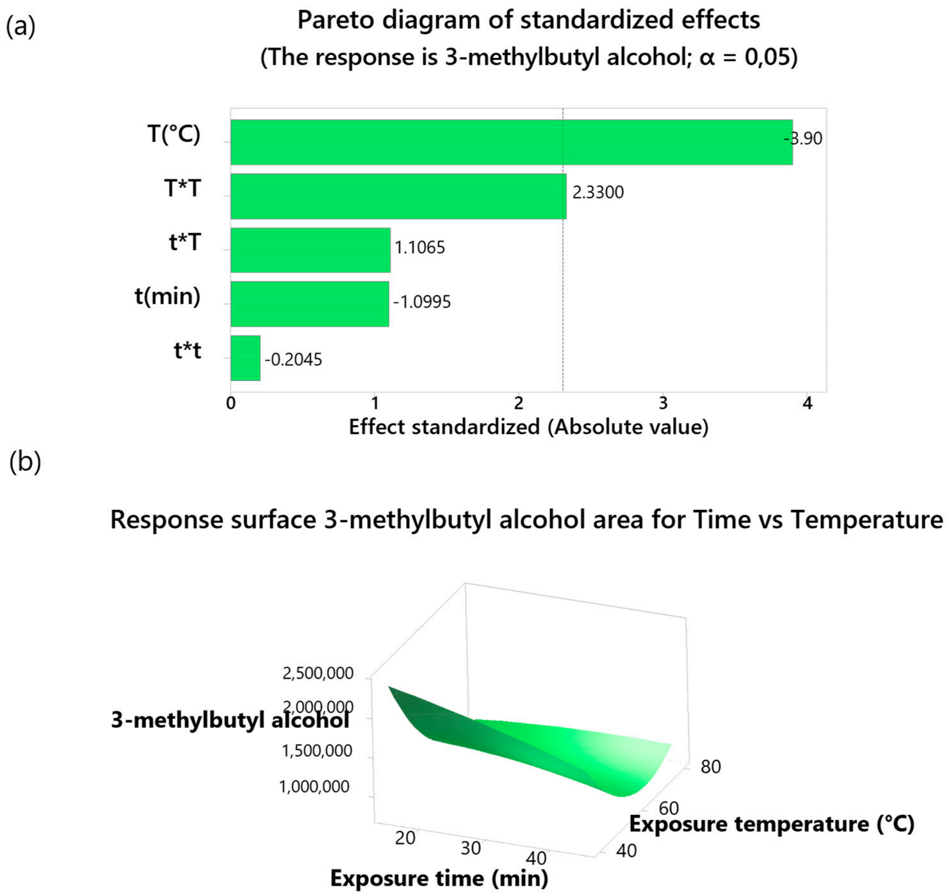

Figure S3. a) Pareto plot of standardized effects for 3-methylbutyl alcohol and b) area response surface of isoamyl alcohol over time vs. extraction temperature.

(a)

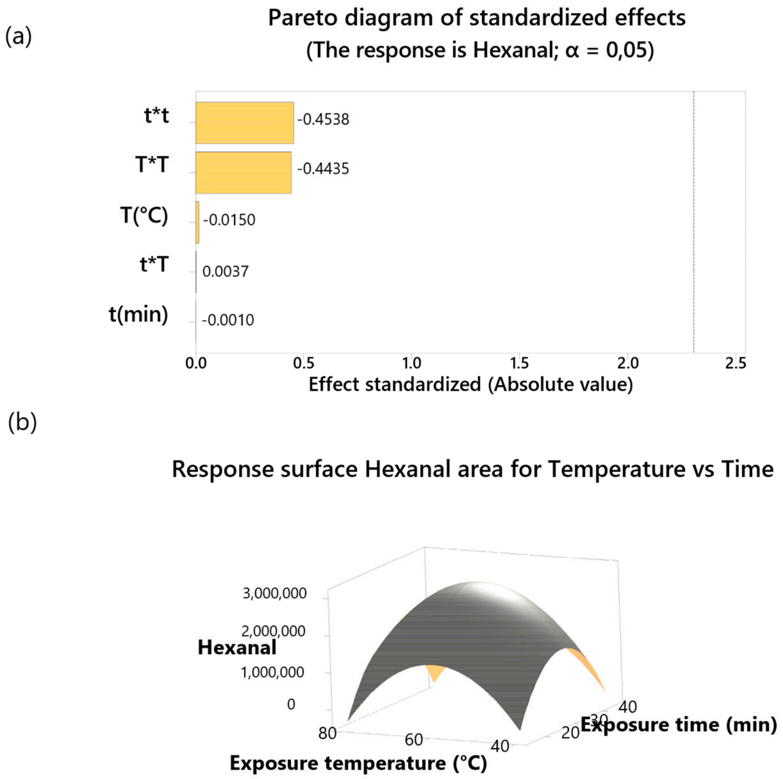

Figure S4. a) Pareto plot of standardized effects for hexanal and b) hexanal area response surface for time vs. extraction temperature.

(a)

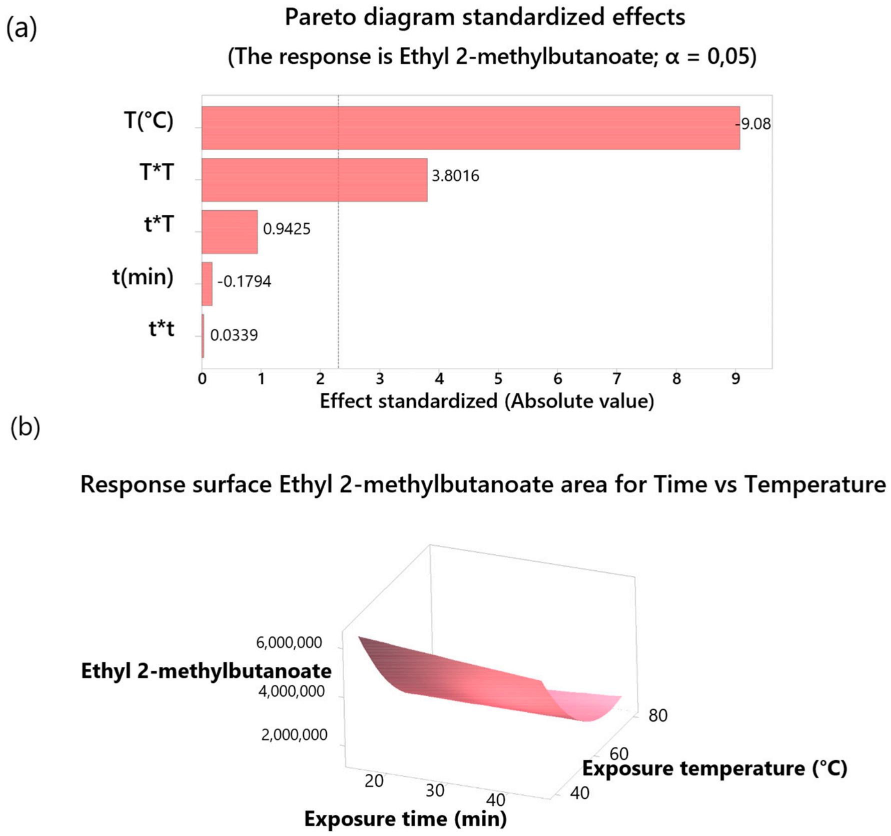

Figure S5. a) Pareto plot of standardized effects for ethyl 2-methylbutanoate and b) area response surface of ethyl 2-methylbutanoate over time vs. extraction temperature.

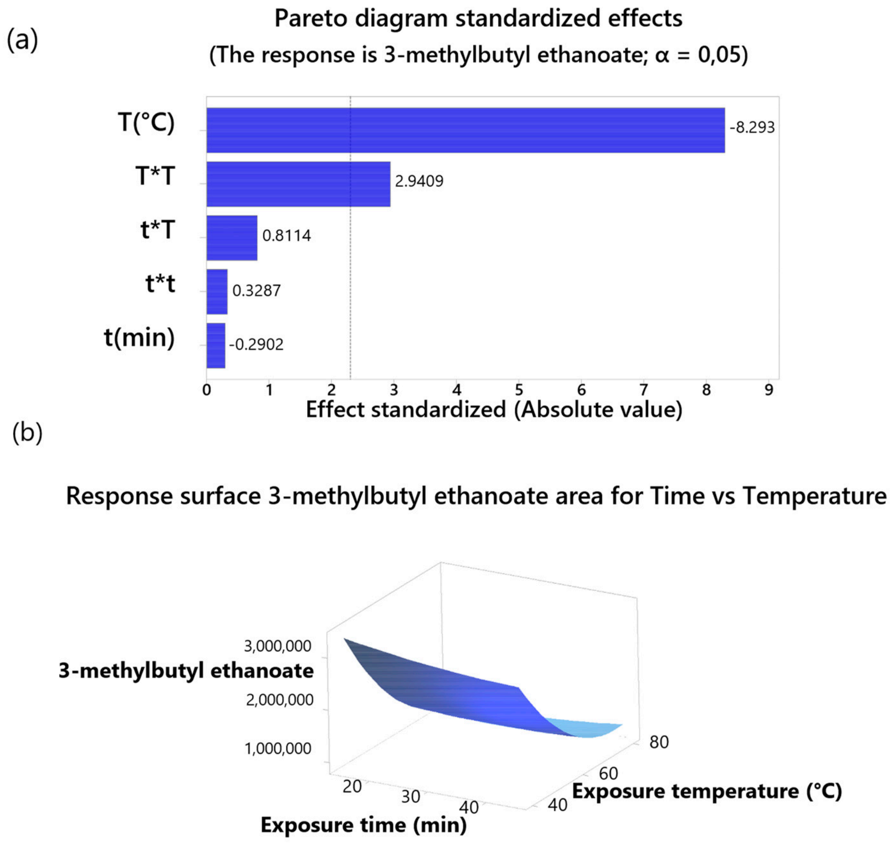

Figure S6. a) Pareto plot of standardized effects for 3-methylbutyl ethanoate and b) area response surface of isoamyl acetate for time versus extraction temperature.

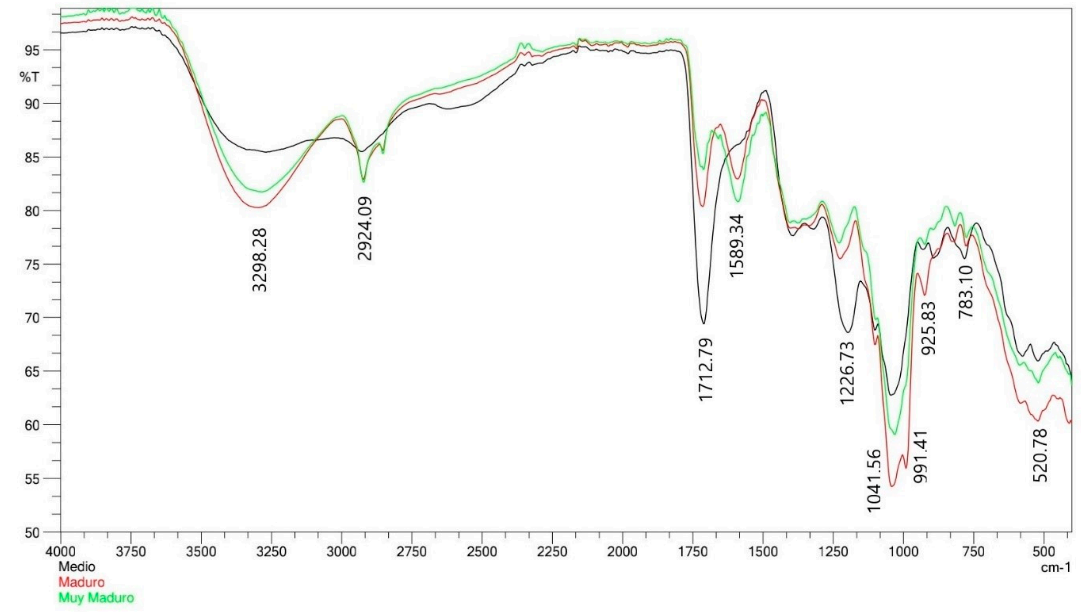

Figure S7. FTIR spectra of ripening stages of copoazu. Medium-ripening stage (black), ripe (red), and very ripe (green).

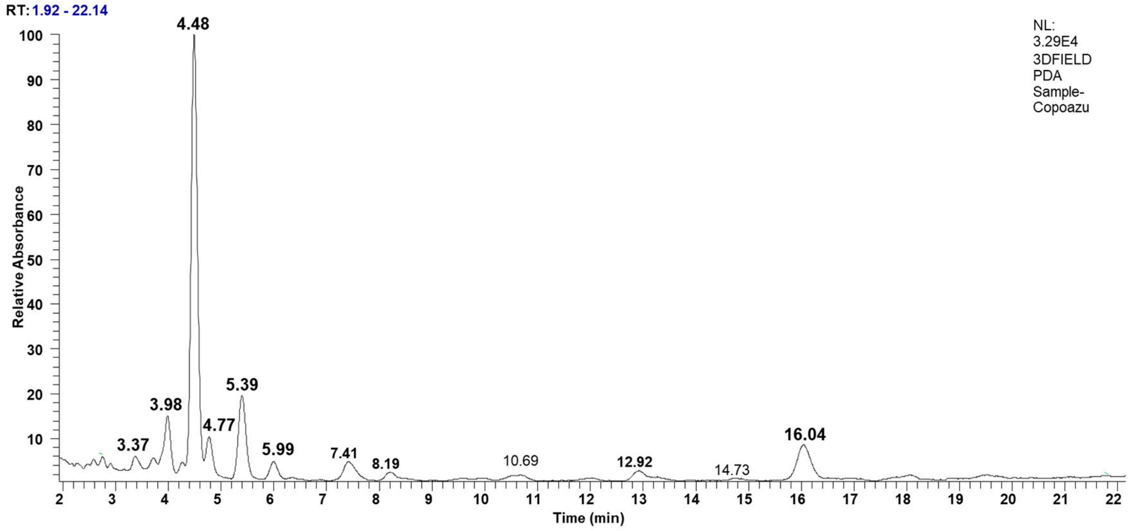

Figure S8. HPLC-DAD chromatogram of carotenoids in copoazu.

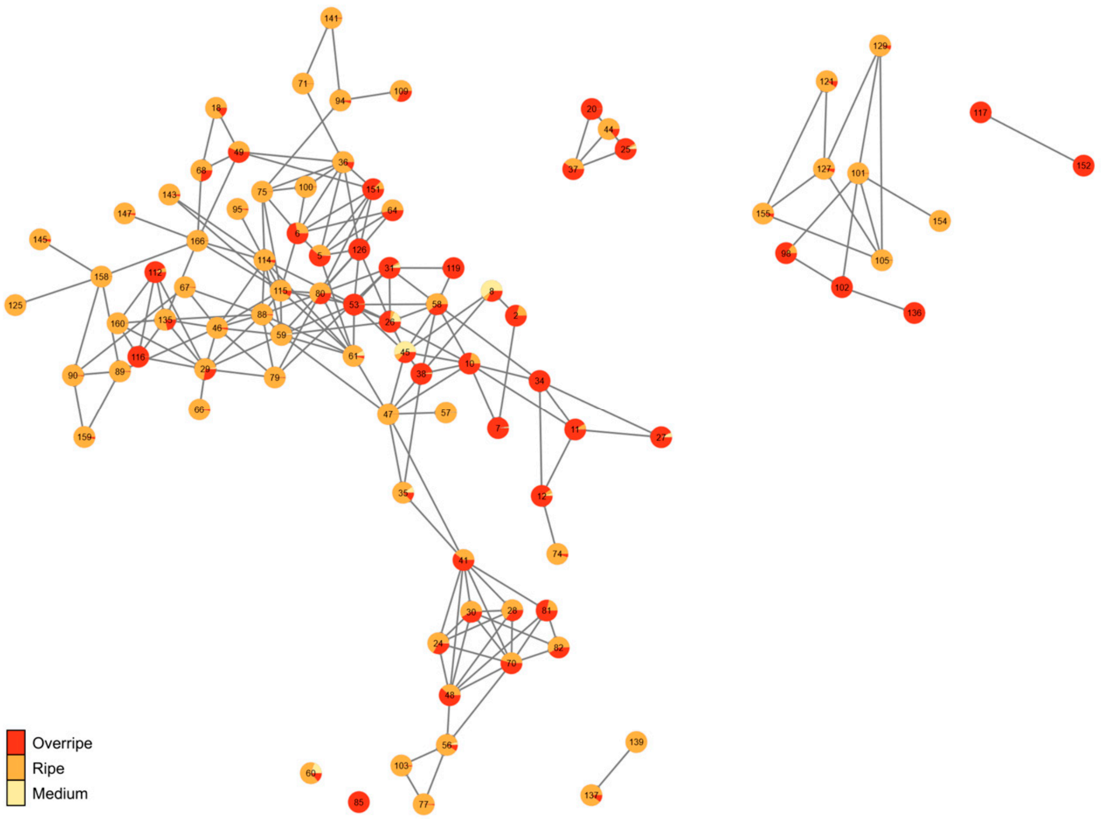

Figure S9. Molecular network from volatilomic profile of the copoazu in three ripening stages. In the molecular network shown the representation of the absolute intensity of the mass spectra at each stage of maturation according to compounds.

Table S1. 2 × 3 factorial design.

Run	Exposure Time (min)	Exposure Temperature (°C)
1	30	60
2	30	60
3	45	40
4	30	60
5	15	40
6	15	80
7	45	80
8	30	80
9	45	60
10	30	60
11	15	60
12	30	60
13	30	60
14	30	40

Table S2. Physicochemical parameters of copoazu fruit at different stages of ripening

Test	Maduration stage		
	**Medium**	**Ripe**	**Overripe**
**SST (%)**	4,5	10,5	12
**pH**	3,40	3,47	4,04

Table S3. Tentative identification of seven copoazu carotenoids.

**N°**	**Compound**	**RT (min)**	**ʎ max (nm)**
1	(all-E)Zeaxanthin	3.98	423, 443, 468
2	(all-E)-lutein 3′-O-palmitate	4.49	443, 470
3	(all-E)Luteoxathin	4.78	399, 423, 450
4	(all-E)-Antheraxanthin	5.41	425, 447, 472
5	(13Z)-Lutein	5.99	416, 438, 466
6	(all-E)-Neoxanthin	7.41	423, 436, 466
7	(9Z)β-caroteno	16.00	424, 448, 471

RT: retention time, ʎ max: Absorbance maxima
